# Microwave-Assisted Synthesis of Flower-like MnMoO_4_ Nanostructures and Their Photocatalytic Performance

**DOI:** 10.3390/ma17071451

**Published:** 2024-03-22

**Authors:** Muthamizh Selvamani, Arulvarman Kesavan, Arunachalam Arulraj, Praveen C. Ramamurthy, Mostafizur Rahaman, Saravanan Pandiaraj, Muthu Thiruvengadam, Elisban Juani Sacari Sacari, Elmer Marcial Limache Sandoval, Mangalaraja Ramalinga Viswanathan

**Affiliations:** 1 Department of Physiology, Saveetha Dental College & Hospitals, Saveetha Institute of Medical & Technical Sciences, Saveetha University, Chennai 600077, Tamil Nadu, India; muthamizh23@ymail.com; 2 Department of Physics & Nanotechnology, SRM Institute of Science & Technology, Kattankulathur 603203, Tamil Nadu, India; 3 Departamento de Electricidad, Facultad de Ingeniería, Universidad Tecnológica Metropolitana (UTEM), Macul, Santiago 7800002, Chile; arul@utem.cl; 4 Department of Materials Engineering, Indian Institute of Science, Bangalore 560012, Karnataka, India; praveen@iisc.ac.in; 5 Department of Chemistry, College of Science, King Saud University, P.O. Box 2455, Riyadh 11451, Saudi Arabia; mrahaman@ksu.edu.sa; 6 Department of Self-Development Skills, King Saud University, P.O. Box 2455, Riyadh 11451, Saudi Arabia; psaravanan.c@ksu.edu.sa; 7 Department of Applied Bioscience, College of Sanghuh Life Science, Konkuk University, Seoul 05029, Republic of Korea; thiruv30@gmail.com; 8 Centro de Energías Renovables de Tacna, Facultad de Ciencias, Universidad Nacional Jorge Basadre Grohmann, Avenida Miraflores S/N, Ciudad Universitaria, Tacna 23003, Peru; esacaris@unjbg.edu.pe; 9 Grupo de Investigación HIDROCIENCIA, Facultad de Ciencias de la Salud, Universidad Privada de Tacna, Av. Jorge Basadre Grohmann S/N Pocollay, Tacna 23003, Peru; 10 Faulty of Engineering and Sciences, Universidad Adolfo Ibáñez, Diagonal las Torres 2640, Peñalolén, Santiago 7941169, Chile; mangal@uai.cl; 11 Department of Mechanical Engineering, Faculty of Engineering, Karpagam Academy of Higher Education, Coimbatore 641021, Tamil Nadu, India

**Keywords:** MnMoO_4_ flower, photocatalyst, visible light active, environmental

## Abstract

This article describes an affordable method for the synthesis of MnMoO_4_ nanoflowers through the microwave synthesis approach. By manipulating the reaction parameters like solvent, pH, microwave power, and irradiation duration along this pathway, various nanostructures can be acquired. The synthesized nanoflowers were analyzed by using a powder X-ray diffractometer (XRD), field emission scanning electron microscopy (FE-SEM) with energy dispersive X-ray spectroscopy (EDS), Fourier transform infrared spectroscopy (FT-IR), and UV–vis diffuse reflectance spectroscopy (UV–DRS) to determine their crystalline nature, morphological and functional group, and optical properties, respectively. X-ray photoelectron spectroscopy (XPS) was performed for the examination of elemental composition and chemical states by qualitative and quantitative analysis. The results of the investigations demonstrated that the MnMoO_4_ nanostructures with good crystallinity and distinct shape were formed successfully. The synthesized MnMoO_4_ nanoflowers were tested for their efficiency as a photocatalyst in the degradation studies of methylene blue (MB) as model organic contaminants in an aqueous medium under visible light, which showed their photocatalytic activity with a degradation of 85%. Through the band position calculations using the electronegative value of MnMoO_4_, the photocatalytic mechanism of the nanostructures was proposed. The results indicated that the effective charge separation, and transfer mechanisms, in addition to the flower-like shape, were responsible for the photocatalytic performance. The stability of the recovered photocatalyst was examined through its recyclability in the degradation of MB. Leveraging MnMoO_4_’s photocatalytic properties, future studies may focus on scaling up these processes for practical and large-scale environmental remediation.

## 1. Introduction

Poisonous and dangerous substances affect the ecological integrity and ecosystems of the globe, necessitating comprehensive environmental organization and rehabilitation [1]. The persistence, non-biodegradability, and bioaccumulation of numerous biochemical pollutants endanger social wellbeing and reduce financial wellbeing, creating significant problems for environmental groups. The rapid industrial expansion, fueled by unsustainable technical breakthroughs, can result in industrial wastewater, which is generated in significant quantities worldwide each year, increasing chemical risks [2]. These substances have been strongly connected to significant consequences and long-term impacts, the majority of which are related to acute water poisoning. To address the quick identification and detection of these problematic substances, proper risk assessment methodologies are required [3]. The contamination of water is caused by a variety of pollutants, including inorganic (salts, caustic metal ions, and metal oxides) and organic (pesticides, fertilizers, phenol, and dyes) pollutants, agricultural runoff, and so on [4,5,6]. The methods for removing organic matter can be distinctly separated into three groups: (i) physical, (ii) chemical, and (iii) biological, which include precipitation [7], ion exchange [8], sonochemical degradation [9], adsorption [10], and various advanced oxidation process techniques such as Fenton [11], photo-Fenton [12], photocatalysis [13], etc.

The degradation of environmental contaminants using photocatalysis is an interesting approach. With its capacity to break down different pollutants and convert light energy into chemical energy, photocatalysis has captured the attention of researchers studying possible applications of today’s technological advancements. This opens up promising avenues for the removal of potentially dangerous contaminants from the environment [14,15]. Photocatalysis is the succession of the oxidation and reduction processes caused by electrons and holes stimulated by photons. The photocatalytic performance of the catalysts will eventually be linked with its shape, chemical and surface characteristics, and crystallinity. Hence, using the photocatalysis approach as a strategy for water purification necessitates the active use of UV and visible light, as well as the usage of highly complicated photocatalysts. The development of a photocatalysis system that counteracts the visible and UV light is critical for removing organic pollutants from the wastewater [16,17]. 

Molybdenum (Mo)-based nanomaterials have been largely studied in the field of photocatalytic pollutant degradation. They have been extensively used as environmental remediation materials because of their exceptional performance in photocatalysis. Molybdenum oxides have garnered significant attention in recent times for their potential applications in several domains, such as humidity sensors, magnetic fields, the microwave approach, optical fibers, scintillator materials, photoluminescence devices, catalysis, and electrochemical sensors, owing to their physiochemical characteristics [18,19,20,21]. Adding certain precious metals to the oxides can enhance their optical, catalytic, and sensing properties. In recent decades, several metal molybdates with various shapes and characteristics have been documented [22]. Molybdates are compounds containing molybdenum oxy anions in which molybdenum has the formal oxidation number of +6. Molybdates are a significant group of transition metal oxides. They possess an extremely large bivalent cation with an ionic radius >0.99 for scheelite structures, such those of Ba, Sr, Pb, or Ca. In this way, the binary metal oxide manganese molybdate (MnMoO_4_) has received a lot of research interest recently because of its affordability, viability as oxidation states, improved electrical conductivity, and good thermal and chemical stability, allowing it to achieve superior electrochemical performance [23,24]. 

Additionally, a wide range of rich redox reactions are offered by the metal ions in binary metal oxides. Among the binary metal oxides, MnMoO_4_ has attracted a lot of attention due to its low cost, environmental friendliness, excellent electrical conductivity, broad working voltage window, and remarkable stability. Furthermore, MnMoO_4_ exhibits outstanding qualities including natural abundance, low toxicity, and a stable crystal structure. Its enormous theoretical specific capacitance and strong electrical conductivity make it environmentally beneficial. The binary metal naturally has a wide operating voltage window and a high retention capacity for energy storage devices [25]. 

In the present investigation, the microwave synthesis methodology was utilized for the production of MnMoO_4_. The morphology of synthesized MnMoO_4_ was confirmed to be flower-like structures. The structural and optical properties of synthesized MnMoO_4_ were confirmed and the phase validation was performed. The synthesized catalyst was utilized for the degradation of methylene blue (MB) through the photocatalytic degradation process as a representative organic toxin. 

## 2. Materials and Methods

The basic starting materials used in the synthesis of MnMoO_4_ such as manganese acetate Mn(CH_3_CO_2_)_2_·(H_2_O), ammonium molybdate ((NH_4_)_6_Mo_7_O_24_), and methylene blue (MB) were analytical grade chemicals acquired from SRL, India. Two solvents were utilized in the synthesis process: ethanol and double-distilled water (DDW). 

### 2.1. Synthesis of MnMoO_4_ Flowers

The simple microwave approach was used for producing MnMoO_4_ flowers. The typical methodology involved dissolving 2 mmol of Mn(CH_3_CO_2_)_2_·(H_2_O) in 30 mL of polyethylene glycol and 0.27 mmol of ammonium molybdate in 30 mL of polyethylene glycol separately at 70 °C, with each solution named as solution A and solution B. Both the solutions were added to each other drop by drop. The pH of the reaction medium was brought to 11 through introducing ethylenediamine; the suspension was subjected to microwave radiation at 750 W for 7 min. The final sample was cleaned multiple times with DDW and ethanol before being oven-dried for 6 h at 90 °C. The resultant powder was ultimately calcined for 2 h at 500 °C to obtain the crystalline MnMoO_4_ nanoflowers.

### 2.2. MnMoO_4_ Nanoflower Investigated with Different Characterizations

The XRD method was used to determine the sample’s crystallinity, and a Rich Siefert 3000 diffractometer was used to measure the sample’s response to Cu-K_α1_ radiation (λ = 1.5406). X-ray photoelectron spectroscopy (XPS) performed by Omicron nanotechnology-ESCA-14, Germany, was employed to define the chemical structure of the manganese molybdate. The FT-IR spectroscopic analysis (using a Perkin-Elmer spectrophotometer) was used to examine the compound’s functional group. For the sample analysis, the KBr pellet technique was employed. The Raman-11 Nano Photon Corporation Instrument, manufactured in Japan, was utilized for the Raman analysis. Using a Perkin-Elmer spectrophotometer, the material’s optical properties were evaluated. By using field emission scanning electron microscopy (FE-SEM), HITACHI SU6600, FEI, Hitachi High-Technologies, Tokyo, Japan, the morphology of manganese molybdate was recorded. 

## 3. Results

### 3.1. Structural Analysis by XRD

**Figure 1:**
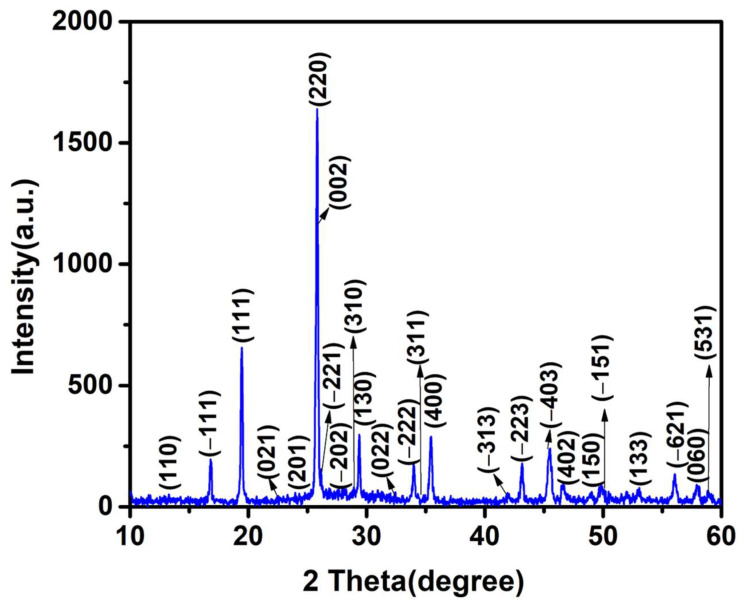
XRD spectrum of synthesized MnMoO_4_ flowers.

Figure 1 displays the synthesized MnMoO_4_ XRD pattern. A diffraction peak was allocated to the space group p2/c of JCPDS Card No. 01-082-2166, which corresponds to the monoclinic structure of MnMoO_4_. It displays the peaks corresponding to (110), (-111), (111), (021), (201), (220), (002), (-221), (-202), (310), (130), (022), (-222), (311), (400), (-313), (-223), (-403), (402), (150), (-151), (133), (-621), (060), and (531) of monoclinic MnMoO_4_. The lattice specifications of MnMoO_4_ are a = 10.49 Å, b = 9.52 Å, c = 7.15 Å, and β = 106.33°. The synthesized material is highly crystalline in nature, as seen through the strong diffraction of MnMoO_4_. Furthermore, there were no further impurity peaks found [26].

### 3.2. XPS Investigation

**Figure 2:**
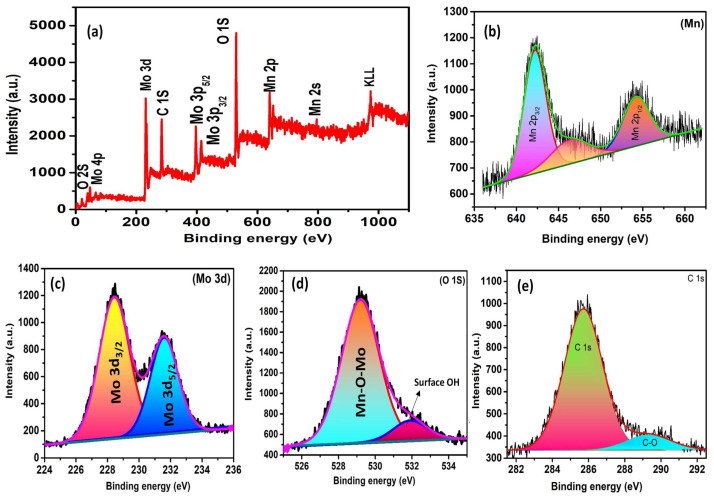
The XPS (**a**) survey spectrum of MnMoO_4_; (**b**–**e**) core-level spectrum of Mn, Mo, O, and C, respectively.

The MnMoO_4_ nanostructures were subjected to XPS examination in order to verify the elements’ oxidation states, binding energies, and chemical composition. The survey XPS spectrum of MnMoO_4_ is displayed in Figure 2a. It displays the occurrence of Mn, Mo, and O in the produced MnMoO_4_. This outcome aligns with the EDAX study. Additionally, the following section discusses the high-resolution XPS spectra of the produced MnMoO_4_ nanoflowers for Mn 2p, Mo 3d, and O 1s. The XPS spectrum of Mn 2p is shown in Figure 2b. Doublet peaks are seen at 641.5 and 653.7 eV, respectively, which correspond to Mn 2p_3/2_ and Mn 2p_1/2_. This indicates that the oxidation state of +2 of the Mn in the sample is present. The Mo 3d XPS spectrum is seen in Figure 2c, with two peaks at 228.4 and 231.5 eV, respectively, that belong to Mo 3d. The measured Mo 3d_3/2_ and Mo 3d_5/2_ doublets’ separation was found to be 3.1 eV, corresponding to the Mo +6 oxidation state [27,28]. The O 1s peak has a prominent component centered at 529.2 eV with a low binding energy; this is indicative of the oxide that forms O^2-^ with components of manganese and molybdenum (Mn–O–Mo), and 531.9 eV corresponds to the surface hydroxyl group, as shown in Figure 2d [29]. The C1s peak is fitted with two peaks at 285.6 and 289.3 eV which belong to C 1s and C-O, respectively [30]. Further, by referring to the previous literature, quantification of the synthesized catalyst MnMoO_4_ is calculated. Mn, Mo, and O elements are displayed along with an error bar in atomic % from the high-resolution XPS spectra; the relevant values are provided in Table 1 [31].

### 3.3. Structural and Optical Properties 

**Figure 3:**
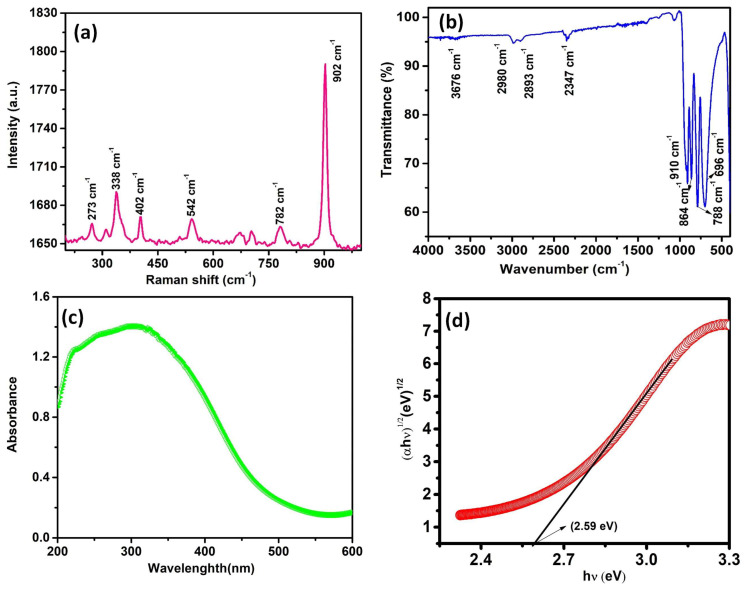
(**a**–**d**) The Raman, FT-IR, and DRS-UV spectra corresponding to the bandgap of the prepared MnMoO_4_ flowers.

Figure 3a displays the synthesized MnMoO_4_ Raman spectrum. The measured bands for the synthesized MnMoO_4_ correspond to the different modes. MnMoO_4_ was found to have bands at 902, 782, 542, 402, 338, and 273 cm^−1^. The Raman peaks at 902 and 782 cm^−1^ are caused by symmetrical stretching of Mo-O-Mo and asymmetrical stretching of υ as Mo=O [32]. The Mo-O-Mo bond, which is due to mutual oxygen atoms of 2 and 3 octahedral MoO_6_, due to symmetric and asymmetric stretching, is confirmed by peaks at 542 and 402 cm^−1^ [33]. The signal that was seen at 273 cm^−1^ is consistent with the double bond O=Mo=O in MnMoO_4_ nanostructures due to wagging. Figure 3b shows the FT-IR spectra of the synthesized MnMoO_4_. The surface hydroxyl O-H bond vibration is shown by a wide band seen at 3436 cm^−1^. The H-O-H distortion mode of the surface hydroxyl assembly is represented by the band at 1637 cm^−1^ [34]. Peaks of MnMoO_4_ are seen at around 864 and 910 cm^−1^, which corresponded to the stretching and bending vibration of Mo=O and Mo–O–Mo, respectively. The optical property of produced MnMoO_4_ was examined using UV-vis diffuse reflectance spectroscopy (DRS) and the obtained result is presented in Figure 2c. The robust absorption peak at 303 nm is due to the switch among bands of O 2p and Mn 3d levels in the MnMoO_4_ [35]. The bandgap (E_g_) of the manufactured MnMoO_4_ was calculated through Tauc’s plot (Equation (1)).

(hυα)^1/n^ = A(hυ − E_g_) (1)

The synthesized MnMoO_4_ presented a direct bandgap of 2.59 eV, which is shown in Figure 3c,d [36]. Additionally, the conduction band (CB) and valence band (VB) positions were identified by using Equations (2) and (3):

E_VB_ = X − E^e^ + 0.5E_g_ (2)

E_CB_ = E_VB_ − E_g_ (3)

The conduction band (CB) edge potential is denoted by E_CB_, the bandgap energy is represented by E_g_, the valence band (VB) edge potential is represented by E_VB_, and the electronegativity of MnMoO_4_ is represented by X (5.94 eV). The E_CB_ value for the MnMoO_4_ flowers is determined to be −0.305 eV using these calculations. Furthermore, the E_VB_ value is estimated as 1.26 eV. The oxygen vacancies’ role in the valence band edge increasing and the conduction band edge reducing is verified by these computations. To enable effective photocatalytic activity, the oxygen vacancies ideally stimulate the creation of sub-bands (below but near to CB) that both limit electron–hole recombination and function as amplifiers for the shift of electrons.

## 4. Morphological Analysis

**Figure 4:**
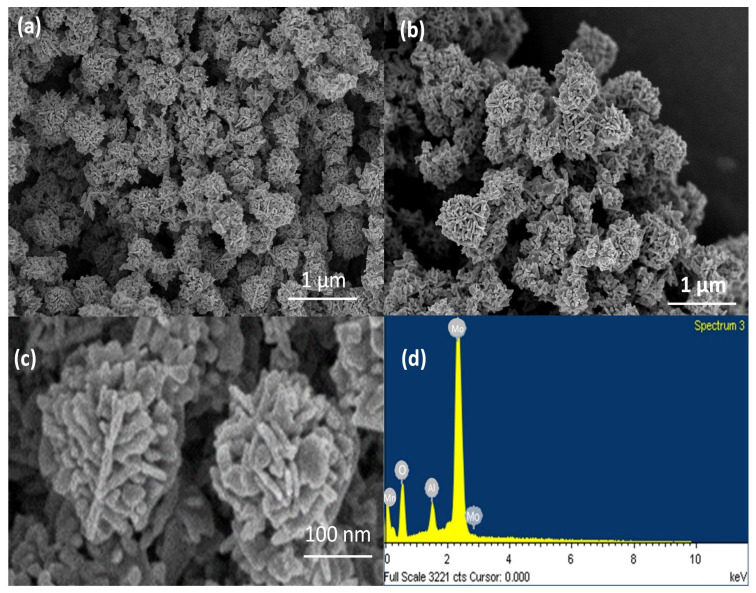
(**a**–**c**) The FE-SEM images of MnMoO_4_ and (**d**) the corresponding EDS.

The FE-SEM images of MnMoO_4_ revealed the formation of flower-like structures, as shown in Figure 4a–d. These nanoflowers exhibited a high degree of MnMoO_4_ nanopetal arrangement to form a uniform flower-like morphology with size of 1 µm. The MnMoO_4_ nanoflowers are assembled from discrete nanopetals with a thickness of a nanometer [37]. This hierarchical flower has more dense petals which may favor the photocatalytic activity due to the involvement of a greater number of nanopetals at a time. The EDS analysis (Figure 4d) confirmed the elemental composition of the MnMoO_4_ flowers. The spectrum displayed peaks corresponding to manganese (Mn), molybdenum (Mo), and oxygen (O), respectively. 

## 5. Photodegradation of MB in the Presence of MnMoO_4_

**Figure 5:**
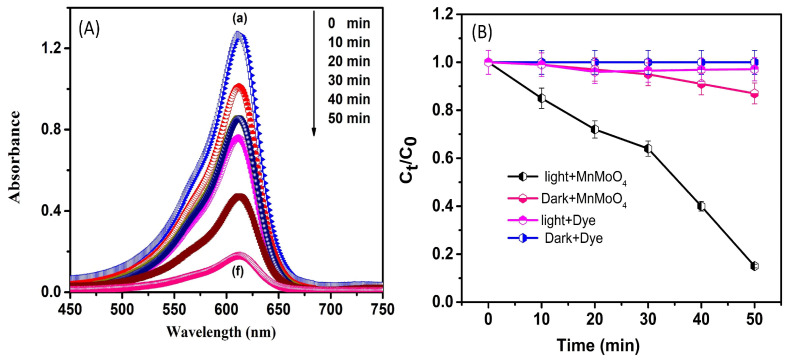
(**A**) The absorption spectra of methylene blue degradation with MnMoO_4_ flowers from 0 to 50 min (represented by (a) to (f)) during photocatalysis, and (**B**) C/C_0_ vs. time plot of methylene blue.

**Figure 6:**
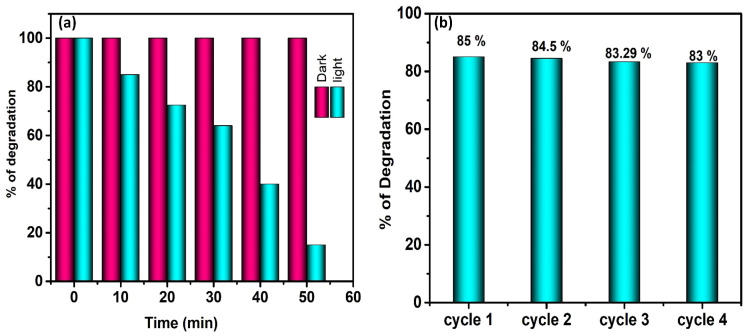
(**a**) The degradation efficacy % with respect to time; (**b**) the cycling test of the photocatalytic degradation of pollutants on MnMoO_4_ flowers.

Figure 5A portrays the photocatalytic action of the manufactured MnMoO_4_ flowers studied regarding the breakdown of methylene blue (MB) dye in the presence of visible light irradiation. Two types of control experiments were conducted by taking a blank dye (MB) solution of 1 × 10^−5^ and a dye with MnMoO_4_ in dark conditions to compare the photocatalytic action of the material in the existence of light. The typical procedure of combining dye with a catalyst in the presence of light involves the addition of 100 mL of a 1 × 10^−5^ M MB dye solution to a 250 mL beaker along with 50 mg of the catalyst (MnMoO_4_). The mixture of pollutant and catalyst was obtained in the dark, preventing contact with light, to achieve the adsorption–desorption balance of the photocatalyst and dye [38,39]. Among the above two reaction mixtures, the pure dye solution and the dye with the catalyst were maintained in the dark, the dye was set with light, and the material with dye was exposed to the visible light after half an hour while being constantly stirred with magnetic stirrer. At regular intervals of every 10 min, all of the three sets of reaction mixture were collected by using a syringe filter to avoid sample contamination during UV-vis analysis. Figure 5B shows the C/C_0_ vs time plot of methylene blue in the dark, methylene blue in light, and methylene blue in the dark and light exposure condition in the presence of the photocatalyst. The color of the MB solution changes from dark blue to leucomethylene blue (colorless) as the MB decrease happens, which demonstrates the photodegradation plot of the pollutant molecules proposed by observing the decrease in the pollutant content under solar light. Firstly, the degradation of the MB solution was estimated in the absence of light to observe the chemical progression in the absence of light. Upon illumination, the pollutant concentration was gradually removed through photoreaction; the concentration in the dye deprived of any catalyst was noted to be 2.9% at 50 min, and the dye with the catalyst in the dark condition displayed the adsorption of the pollutant on the outer layer of catalyst, with the concentration being 13% at 50 min [40]. As the photocatalyst reaction proceeds with the MnMoO_4_ flowers as the photocatalyst, it was seen that the absorption maximum of the dye peaks at 665 nm, reducing gradually at 50 min, with 85% of the dye observed, as shown in Figure 6a. The photocatalytic property of the present catalyst is matched with that previously stated in the literature and currently proposed in Table 2.

### Cycling Test to Remove Pollutants Using Photocatalysis

**Figure 7:**
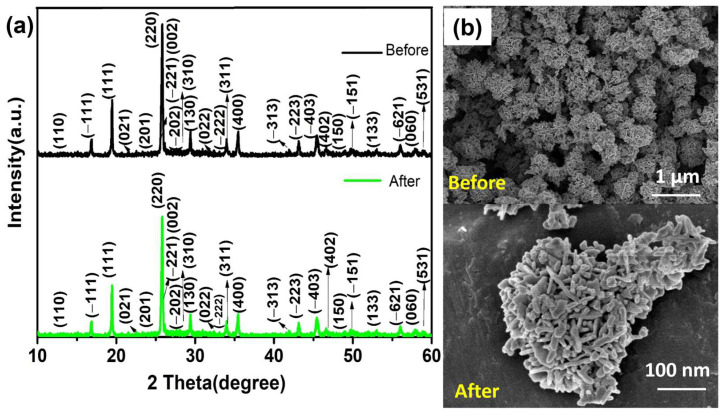
(**a**) The XRD patterns and (**b**) the FE-SEM analysis of MnMoO_4_ flowers synthesized and after catalytic reaction.

The photodegradation and stability of MnMoO_4_ flowers toward MB dye was examined under identical reaction conditions. The MnMoO_4_ photocatalyst was separated after the degrading process and given a thorough deionized water and ethanol wash. Before using the recovered catalyst in a second run, it was desiccated at 100 °C in a hot-air oven. The MB dye deterioration during the four runs is displayed in Figure 6b. In the consecutive four cycles with MnMoO_4_ flowers, the MB dye degradation percentages were 85.00, 84.50, 83.29, and 83.00, respectively, with a 50 min reaction runtime. The MnMoO_4_ flowers deterioration efficiency was not altered significantly, even after the consecutive four tests. Stability and reusability are essential components for a photocatalyst to be used in practical applications. The structural consistency of the MnMoO_4_ flowers was tested later through X-ray diffraction analysis and morphological studies after four cycles; the obtained results are presented in Figure 7a,b. These results demonstrate that except from a decline in peak strength, morphology, and repeatable action, there are no observable structural changes during the photocatalytic processes. Consequently, it is possible to utilize wastewater management schemes for a long time.

## 6. Conclusions

The microwave-assisted synthesis process was used to create the flower-like structured MnMoO_4_. XRD examination was utilized to confirm the monoclinic phase structure and the crystalline environment of the synthesized MnMoO_4_ flowers. Further, the chemical bonding of the molecule and the functional group identification of the MnMoO_4_ flowers were characterized via XPS, FTIR, and Raman analyses. The direct bandgap of 2.3 eV of the MnMoO_4_ was determined from the UV vis-DRS analysis and the flower-like structured morphology of the MnMoO_4_ was observed from the FE-SEM analysis. Finally, the produced material was studied for cycle stability and photocatalytic degradation effectiveness toward the organic pollutant MB dye with an irradiation time of 50 min with 85% of the degradation efficiency. Further, the stability of the synthesized photocatalyst was assessed by executing four consecutive runs of the photocatalyst experiments. Also, to investigate its durability, the FE-SEM and XRD analyses were employed with the post-experimental photocatalyst samples. The post-experimental samples exhibited almost the same morphology and crystal structure, with only a small decline in their peak intensities. Thus, the synthesized MnMoO_4_ flower-like structured photocatalysts could be highly suitable as applications in wastewater treatment for a longer duration.

## Figures and Tables

**Table 1:** Atomic % of the elements present in MnMoO_4_.

**Elements**	Mn	Mo	O
**Atomic %**	19.54	44.42	36.04

**Table 2:** Comparison with other photocatalysts for the degradation of MB.

Catalyst	Dye	Amount of Catalysts	Degradation Efficiency	Degradation Time	Reference
ZnO/g-C_3_N_4_	MB	100 mg	98%	180 min	[41]
N-doped carbon/CuO-Fe_2_O_3_	MB	100 mg	97.4%	180 min	[42]
N-doped ZnO	MB	100 mg	93.2%	30 min	[43]
Cerium-doped copper aluminate	MB	25 mg	72.49%	90 min	[44]
Cu_2_O/BiVO_4_	MB	50 mg	72.9%	160 min	[45]
MnMoO_4_	MB	50 mg	85%	50 min	Present study

## Data Availability

The data presented in this study are available on request from the corresponding author depending on restrictions, e.g., privacy or ethical concerns. The data are not publicly available due to confidentiality.

## References

[1] Farid A., Michael V., Safwat G. (2023). Melatonin Loaded Poly(lactic-Co-Glycolic Acid) (PLGA) Nanoparticles Reduce Inflammation, Inhibit Apoptosis and Protect Rat’s Liver from the Hazardous Effects of CCL4. Sci. Rep..

[2] Jeyachandran S., Srinivasan R., Ramesh T., Parivallal A., Lee J., Sathiyamoorthi E. (2023). Recent Development and Application of “Nanozyme” Artificial Enzymes—A Review. Biomimetics.

[3] Nikitha M., Elanchezhiyan S.S., Meenakshi S. (2023). Photodegradation of Rhodamine-B in Aqueous Environment Using Visible-Active gCN@CS-MoS Nanocomposite. Environ. Res..

[4] Kheskwani U., Ahammed M.M. (2023). Removal of Water Pollutants Using Plant-Based Nanoscale Zero-Valent Iron: A Review. Water Sci. Technol..

[5] Gayathri P.V., Rayaroth M.P., Aravindakumar C.T., Pillai D., Joseph S. (2023). SUNLIGHT-INDUCED Decontamination of Water from Emerging Pharmaceutical Pollutants Using ZnO Nanoparticles. Chemosphere.

[6] Fang J., Wei S., Gao Y., Zhang X., Cheng Y., Wang J., Ma J., Shi G., Bai L., Xie R. (2023). Character Variation of Root Space Microbial Community Composition in the Response of Drought-Tolerant Spring Wheat to Drought Stress. Front. Microbiol..

[7] Czerwionka K., Wilinska A., Tuszynska A. (2020). The Use of Organic Coagulants in the Primary Precipitation Process at Wastewater Treatment Plants. Water.

[8] Charles J., Bradu C., Morin-Crini N., Sancey B., Winterton P., Torri G., Badot P.-M., Crini G. (2016). Pollutant Removal from Industrial Discharge Water Using Individual and Combined Effects of Adsorption and Ion-Exchange Processes: Chemical Abatement. J. Saudi Chem. Soc..

[9] Mehdizadeh P., Amiri O., Rashki S., Salavati-Niasari M., Salimian M., Foong L.K. (2020). Effective Removal of Organic Pollution by Using Sonochemical Prepared LaFeO3 Perovskite under Visible Light. Ultrason. Sonochem..

[10] Titchou F.E., Zazou H., Afanga H., El Gaayda J., Akbour R.A., Hamdani M. (2021). Removal of Persistent Organic Pollutants (POPs) from Water and Wastewater by Adsorption and Electrocoagulation Process. Groundw. Sustain. Dev..

[11] Shokri A., Fard M.S. (2022). A Critical Review in Fenton-like Approach for the Removal of Pollutants in the Aqueous Environment. Environ. Chall..

[12] Remache W., Ramos D.R., Mammeri L., Boucheloukh H., Marín Z., Belaidi S., Sehili T., Santaballa J.A., Canle M. (2022). An Efficient Green Photo-Fenton System for the Degradation of Organic Pollutants. Kinetics of Propranolol Removal from Different Water Matrices. J. Water Proc. Eng..

[13] Selvamani M., Alsulmi A., Sundaramoorthy A., Vadivel S., Kesavan A.V. (2023). Synthesis of ZnWO_4_ Nanorods: The Photocatalytic Effects on RhB Dye Degradation upon Irradiation with Sunlight Light. J. Mater. Sci. Mater. Electron..

[14] Liu N., Yu J., Zhang H., Zhu J., Liu Q., Chen R., Li Y., Li R., Wang J. (2023). Fe-MMT/WO Composites for Chemical and Photocatalysis Synergistic Reduction of Uranium (VI). Chemosphere.

[15] Etemadi H., Soltani T., Yoshida H., Zhang Y., Telfer S.G., Buchanan J.K., Plieger P.G. (2022). Synergistic Effect of Redox Dual PdO /MnO Cocatalysts on the Enhanced H Production Potential of a SnS/α-FeO Heterojunction via Ethanol Photoreforming. ACS Omega.

[16] Kumar P.S., Biju C.S., Johnson J. (2023). Influence of Annealing on the Structural, Morphological, Photoluminescence and Visible Absorption Properties of Mg Doped CuO Micro Grains. J. Fluoresc..

[17] Gaffar S., Kumar A., Alam J., Riaz U. (2023). Efficient Visible Light-Induced Photocatalytic Degradation of Tetracycline Hydrochloride Using CuFeO and PANI/CuFeO Nanohybrids. Environ. Sci. Pollut. Res. Int..

[18] Tong Y., Zhang Z., Hou Y., Yan L., Chen X., Zhang H., Wang X., Li Y. (2023). Recent Progress of Molybdenum Carbide Based Electrocatalysts for Electrocatalytic Hydrogen Evolution Reaction. Nanoscale.

[19] Xu C., Chang P., Liu Z., Guan L., Wang X., Tao J. (2023). Electrochemical Activated Molybdenum Oxides Based Multiphase Heterostructures with High Hydrogen Evolution Activity in Alkaline Condition. Nanotechnology.

[20] Mitsumoto T., Ashida Y., Arashiba K., Kuriyama S., Egi A., Tanaka H., Yoshizawa K., Nishibayashi Y. (2023). Catalytic Activity of Molybdenum Complexes Bearing PNP-Type Pincer Ligand toward Ammonia Formation. Angew. Chem. Int. Ed. Engl..

[21] Muthamizh S., Sengottaiyan C., Jayavel R., Narayanan V. (2020). Facile Synthesis of Phase Tunable MoO_3_ Nanostructures and Their Electrochemical Sensing Properties. J. Nanosci. Nanotechnol..

[22] Qin L., Huang S., Cheng H. (2023). Catalytic Performance and Mechanism of Bismuth Molybdate Nanosheets Decorated with Platinum Nanoparticles for Formaldehyde Decomposition at Room Temperature. J. Colloid Interface Sci..

[23] Wu T.H., Liu Y.S., Hong C.T., Hou B.-W. (2023). Binary and Nanostructured NiMn Perovskite Fluorides as Efficient Electrocatalysts for Urea Oxidation Reaction. J. Colloid Interface Sci..

[24] Abbas Q., Khurshid H., Yoosuf R., Lawrence J., Issa B.A., Abdelkareem M.A., Olabi A.G. (2023). Engineering of Nickel, Cobalt Oxides and Nickel/cobalt Binary Oxides by Electrodeposition and Application as Binder Free Electrodes in Supercapacitors. Sci. Rep..

[25] Wu Q., Chen L., Kuo D.-H., Li P., Abdeta A.B., Zelekew O.A., Lin J., Chen X. (2023). Sulfur Substitution and Defect Engineering in an Unfavored MnMoO Catalyst for Efficient Hydrogen Evolution under Visible Light. ACS Appl. Mater. Interfaces.

[26] Tamboli M.S., Patil S.A., Tamboli A.M., Patil S.S., Truong N.T.N., Lee K., Praveen C.S., Shrestha N.K., Park C., Kale B.B. (2022). Polyaniline-Wrapped MnMoO as an Active Catalyst for Hydrogen Production by Electrochemical Water Splitting. Dalton Trans..

[27] Jabeen S., Kumar P., Samra K.S. (2023). Boosting the Electrochemical Characteristics of MnMoO_4_ Nanoparticles for Supercapacitor Applications. J. Appl. Electrochem..

[28] Venkatesh K., Rajakumaran R., Chen S.-M., Karuppiah C., Yang C.-C., Ramaraj S.K., Ali M.A., Al-Hemaid F.M.A., El-Shikh M.S., Almunqedhi B.M.A. (2021). A Novel Hybrid Construction of MnMoO Nanorods Anchored Graphene Nanosheets; an Efficient Electrocatalyst for the Picomolar Detection of Ecological Pollutant Ornidazole in Water and Urine Samples. Chemosphere.

[29] Muthamizh S., Suresh R., Giribabu K., Manigandan R., Praveen Kumar S., Munusamy S., Narayanan V. (2015). MnWO_4_ Nanocapsules: Synthesis, Characterization and Its Electrochemical Sensing Property. J. Alloys Compd..

[30] Ranjith K.S., Ezhil Vilian A.T., Ghoreishian S.M., Umapathi R., Hwang S.-K., Oh C.W., Huh Y.S., Han Y.-K. (2022). Hybridized 1D-2D MnMoO-MXene Nanocomposites as High-Performing Electrochemical Sensing Platform for the Sensitive Detection of Dihydroxybenzene Isomers in Wastewater Samples. J. Hazards Mater..

[31] Wang X., Gao Z., Wang C., Guo X., Sun Y., Jia Y., Tao X. (2020). Design, Growth, and Characterization of YMoO Crystals for Raman Laser Applications. RSC Adv..

[32] Wu Q., Gao Z., Wu Z., Li C., Tian X., Zhao P., Wang Z., Sun Y., Xia S., Tao X. (2020). Generation of a Simultaneous Orthogonally Polarized Dual-Wavelength Raman Laser with Power Ratio Tunability by a Single Hexagonal Crystal: CsTeMoO. Opt. Lett..

[33] Ajam M., Ehteshami M., Boudaghpour S., Mirbagheri S.A., Kafil M. (2022). Tetracycline Removal from Aqueous Solution by Biochar Derived from Algae and Modified with MnMoO: Effects of Operating Parameters, Isotherm, Kinetic, and Thermodynamic Study. Int. J. Phytoremediat..

[34] da Silveira Lacerda L.H., San-Miguel M.A. (2022). Unraveling the MnMoO_4_ Polymorphism: A Comprehensive DFT Investigation of α, β, and ω Phases. J. Mater. Sci..

[35] Ghosh D., Giri S., Moniruzzaman M., Basu T., Mandal M., Das C.K. (2014). α MnMoO_4_/graphene Hybrid Composite: High Energy Density Supercapacitor Electrode Material. Dalton Trans..

[36] Chandhana J.P., Punnakkal N., Vasu S.P., Pradeep A., Nair B.G., Babu T.G.S. (2023). Zirconium Copper Oxide Microflowers Based Non-Enzymatic Screen-Printed Electrochemical Sensor for the Detection of Glucose in Saliva, Urine, and Blood Serum. Mikrochim. Acta.

[37] Orudzhev F., Sobola D., Ramazanov S., Částková K., Papež N., Selimov D.A., Abdurakhmanov M., Shuaibov A., Rabadanova A., Gulakhmedov R. (2023). Piezo-Enhanced Photocatalytic Activity of the Electrospun Fibrous Magnetic PVDF/BiFeO_3_ Membrane. Polymers.

[38] Garg A., Chauhan A., Agnihotri C., Singh B.P., Mondem V., Basu S., Agnihotri S. (2023). Sunlight Active Cellulose/g-CN/TiOnano-Photocatalyst for Simultaneous Degradation of Methylene Blue Dye and Atenolol Drug in Real Wastewater. Nanotechnology.

[39] Abdrabou D., Ahmed M., Hussein A., El-Sherbini T. (2023). Photocatalytic Behavior for Removal of Methylene Blue from Aqueous Solutions via Nanocomposites Based on GdO/CdS and Cellulose Acetate Nanofibers. Environ. Sci. Pollut. Res. Int..

[40] Dao T.D., Han G., Arai N., Nabatame T., Wada Y., Hoang C.V., Aono M., Nagao T. (2015). Plasmon-Mediated Photocatalytic Activity of Wet-Chemically Prepared ZnO Nanowire Arrays. Phys. Chem. Chem. Phys..

[41] Ngullie R.C., Alaswad S.O., Bhuvaneswari K., Shanmugam P., Pazhanivel T., Arunachalam P. (2020). Synthesis and Characterization of Efficient ZnO/g-C3N_4_ Nanocomposites Photocatalyst for Photocatalytic Degradation of Methylene Blue. Coatings.

[42] Ren B., Miao J., Xu Y., Zhai Z., Dong X., Wang S., Zhang L., Liu Z. (2019). A Grape-like N-Doped Carbon/CuO-Fe_2_O_3_ Nanocomposite as a Highly Active Heterogeneous Fenton-like Catalyst in Methylene Blue Degradation. J. Clean. Prod..

[43] Prabakaran E., Pillay K. (2019). Synthesis of N-Doped ZnO Nanoparticles with Cabbage Morphology as a Catalyst for the Efficient Photocatalytic Degradation of Methylene Blue under UV and Visible Light. RSC Adv..

[44] Kirankumar V.S., Mayank N., Sumathi S. (2019). Photocatalytic Performance of Cerium Doped Copper Aluminate Nanoparticles under Visible Light Irradiation. J. Taiwan Inst. Chem. Eng..

[45] Min S., Wang F., Jin Z., Xu J. (2014). Cu_2_O Nanoparticles Decorated BiVO_4_ as an Effective Visible-Light-Driven p-n Heterojunction Photocatalyst for Methylene Blue Degradation. Superlattices Microstruct..

